# Bupropion-Induced Severe Leukocytosis

**DOI:** 10.7759/cureus.11083

**Published:** 2020-10-21

**Authors:** Taranjeet S Jolly, Jatinder Singh, Raman Baweja

**Affiliations:** 1 Psychiatry and Behavioral Sciences, Penn State Health Milton S. Hershey Medical Center, Hershey, USA; 2 Psychiatry, Penn State Health Milton S. Hershey Medical Center, Hershey, USA

**Keywords:** bupropion, leukocytosis, emergency, anti depressant, overdose, hematology

## Abstract

Bupropion hydrochloride is a norepinephrine-dopamine reuptake inhibitor approved for the treatment of depression and smoking cessation. Although cardiovascular and hematological side effects are common with bupropion along with weight loss and headache, hematological side effects are rarely reported other than few post marketing studies. Here we present the case of a 47-year-old white female who presented with significant leukocytosis after bupropion overdose.

## Introduction

Bupropion hydrochloride is a norepinephrine-dopamine reuptake inhibitor approved for the treatment of depression and smoking cessation. Bupropion is known to cause cardiovascular side effects such as changes in blood pressure, increased risk for seizures, dermatological and rare hematological side effects such as ecchymosis, anemia, leukocytosis, leukopenia, lymphadenopathy, pancytopenia, and thrombocytopenia which are reported in post marketing studies [1-3]. The extent of leukocytosis in post marketing studies was not reported. Here we present the case of a 47-year-old female who presented with significant leukocytosis after bupropion overdose.

## Case presentation

This is the case of a 47-years-old white female, with a history of major depressive disorder and generalized anxiety disorder, who presented with seizure and altered mental sensorium after eight hours of an intentional overdose on bupropion extended release 4500 mg and trazodone 400 mg. Patient had no prior medical or surgical history. She was immediately moved to the intensive care unit from the emergency room after getting activated charcoal, basic lab draws, and starting of intravenous fluids. On physical examination, she was afebrile and presented with sinus tachycardia, hypertension (blood pressure 190/76 mmHg) and dilated pupils. EKG was noted to be normal other than sinus tachycardia. No signs of urinary tract infection were present. Neuro-imaging of head and neck was normal. Urine analysis and urine drug screen was negative. White blood cell (WBC) count on admission was 30,800 and absolute neutrophil count (ANC) was 30.2. A lumbar puncture was attempted to rule out central nervous system (CNS) infectious etiology but was aborted given the patient's agitation. Patient was treated empirically with broad-spectrum antibiotics. The WBC count trended down to 26,000 by day 2 and mid to low 20,000 by day 3 and 4, respectively. Patient returned back to baseline in terms of her WBC and ANC count by week two in the hospital. Patient was seen by psychiatry in the consultation-liaison service, and was subsequently moved to the psychiatry unit upon getting cleared from the medical floor. Patient was started on sertraline and was optimized to 100 mg/day and was re-started on trazodone 50 mg to address insomnia. Patient was stabilized and was transferred with outpatient psychiatry and therapy follow up.

## Discussion

Bupropion is an antidepressant of the aminoketone class; it is chemically unrelated to tricyclic, tetracyclic, selective serotonin reuptake inhibitor or other known antidepressant agents. Bupropion is commonly associated with side effects such as tachycardia, weight loss, headache, insomnia, nausea, and abdominal pain. Hematological side effects are rare but have been reported in post marketing study. To the best of our knowledge, this is the first case of severe leukocytosis after bupropion overdose. 

There are various explanations of leukocytosis in this case including physiological stress, secondary to seizure activity, infectious etiology, or secondary to bupropion overdose. Leukocytosis secondary to physiological stress usually ranges between 11,000-30,000 per mm3 and lasts for a few hours [4,5]. Similarly, leukocytosis secondary to seizure is also transient with the level usually trending down within 6-9 hours and are not associated with significant elevation of WBC counts (>30,000 per mm3) [6]. In contrast, in this case, the WBC count remained elevated to mid to low 20,000s on day 3 and 4. Additionally, leukocyte count secondary to seizure activity positively correlates with the length of a seizure episode, whereas negatively correlates with the time lapse between the seizure onset and the blood draw. Thus, the longer the seizure and quicker the blood draw, the higher the WBC count [7]. It is very unlikely that leukocytosis, in this case, was secondary to infectious etiology as the patient was afebrile and the blood culture was negative. The possibility of CNS infection cannot be completely ruled out, however, the probability is very low because of normal neuroimaging, normal examination (no neck rigidity), and acute onset after the recent overdose. There is a slight possibility that trazodone can have contributed, however, there are no previous cases of leukocytosis happening from trazodone and since the relative dose of trazodone used by the patient was low, the odds of it leading to such severe leukocytosis seem bleak. While some of the autonomic instability noted initially can be attributed to the nor-epinephrine and dopaminergic action of bupropion, for which is sometimes used off label to treat attention deficit hyperactivity disorder (ADHD), the hematological side effects can still not be explained as well as some of the other side effects such as cardiovascular, neurological or dermatological noted in medications with a predominantly nor-epinephrine or dopaminergic action [8].

Thus, bupropion overdose seems to be the most probable cause of leukocytosis. However, it is unknown how bupropion could lead to such severe leukocytosis. This is the first clinical report of significant leukocytosis following bupropion overdose. Although hematological complications are rare with bupropion, a clinician should be mindful especially in case of bupropion toxicity.

## Conclusions

It is important to be mindful of rare and less documented side effects of psychotropic medications while prescribing it to patients. Although leukocytosis in the context of bupropion is noted only in some post marketing studies, physicians need to be mindful of this side effect along with other less documented hematological side effects with bupropion and antidepressants in general.
